# RNA epitranscriptomic regulation of tumor immune evasion: mechanisms, context-dependent roles, and therapeutic implications

**DOI:** 10.3389/fimmu.2026.1857099

**Published:** 2026-07-08

**Authors:** Yanni Ma, Wenzhi Deng, Xiulin Jiang, Yuxing Zhu

**Affiliations:** 1 Department of Lymphoma and Hematology, Hunan Cancer Hospital, Changsha, China; 2 The Affiliated Cancer Hospital of Xiangya School of Medicine, Central South University, Changsha, China; 3 Department of Pathology, The Third Xiangya Hospital of Central South University, Changsha, China; 4 College of Life Science, University of Chinese Academy of Sciences, Beijing, China; 5 Department of Oncology, The Third Xiangya Hospital of Central South University, Changsha, China

**Keywords:** biomarkers, cancer, immune checkpoint, immunotherapy, precision medicine, RNA epitranscriptomics, tumor immune evasion, tumor microenvironment

## Abstract

Tumor immune evasion is a fundamental hallmark of cancer progression and a major barrier to effective immunotherapy. RNA epitranscriptomic modifications have emerged as a critical layer of post-transcriptional regulation that links RNA fate control with tumor immune remodeling. These reversible modifications, including m^6^A, m^5^C, ac^4^C, m¹A, m^7^G, pseudouridine, m^6^Am, Nm, and A-to-I RNA editing, are dynamically regulated by writers, erasers, and readers. By modulating RNA stability, splicing, nuclear export, translation efficiency, degradation, and innate immune recognition, RNA modifications reshape multiple immune-related processes in cancer. Mechanistically, they regulate tumor immune visibility by influencing antigen processing, MHC-I expression, interferon signaling, and dendritic cell-mediated cross-presentation. They also control immune checkpoint expression, particularly the PD-1/PD-L1 axis, inflammatory signaling pathways, immune-cell recruitment and exhaustion, and metabolic immunosuppression within the tumor immune microenvironment. Importantly, the functions of RNA modification regulators are highly context dependent. The same regulator may either promote immune escape or enhance antitumor immunity depending on cancer type, cellular source, target transcript, reader protein, and microenvironmental state. From a clinical perspective, RNA modification-based molecular subtypes, prognostic signatures, and risk-score models show potential for predicting patient prognosis, immune infiltration, and response to immune checkpoint blockade. In parallel, targeting RNA modification regulators, alone or in combination with immunotherapy, radiotherapy, chemotherapy, or targeted therapy, represents an emerging therapeutic strategy. However, clinical translation remains limited by insufficient specificity, tumor heterogeneity, complex crosstalk among RNA modifications, potential toxicity, and delivery barriers. Future studies integrating RNA modification mapping with single-cell, spatial, and multi-omics technologies will be essential to define cell-type-specific regulatory networks and develop precise RNA epitranscriptomic biomarkers and therapies for cancer immunotherapy.

## 1 Introduction

Tumor immune evasion is a fundamental biological hallmark of cancer initiation and progression (1). It arises from the dynamic transition from immune surveillance to immune escape (1). Under normal conditions, the immune system can recognize and eliminate newly transformed tumor cells (1). However, during tumor evolution, cancer cells gradually develop mechanisms to evade immune recognition and destruction. These mechanisms include reduced antigen presentation, upregulation of immune checkpoint molecules, recruitment of immunosuppressive cells, and remodeling of the TIME (2). As a result, tumor cells escape immune control. This process not only weakens intrinsic antitumor immunity but also contributes to resistance and failure of immunotherapy.

In recent years, RNA epitranscriptomics has emerged as an important regulatory layer following DNA methylation and histone modification. It has become a major focus in cancer research (3). This field studies reversible chemical modifications on RNA and their effects on RNA fate and function (4). These include RNA stability, splicing, nuclear export, and translation efficiency (4). With the development of high-throughput sequencing and chemical labeling technologies, a growing number of RNA modifications have been identified (5). Among them, N6-methyladenosine (m^6^A) is the most abundant and well characterized. Other modifications, including 5-methylcytosine (m^5^C), N4-acetylcytidine (ac^4^C), N1-methyladenosine (m^1^A), 7-methylguanosine (m^7^G), and pseudouridine (Ψ), N6,2’-O-dimethyladenosine (m6Am), 2′-O-methylation (Nm) and A-to-I RNA editing, also play important roles in cancer and immune regulation (5–14). RNA modifications are increasingly recognized as key molecular links between tumor cells and the immune system (15, 16). On one hand, they directly regulate the expression of immune checkpoint molecules such as PD-1/PD-L1, cytokines, and genes in critical signaling pathways (17, 18). On the other hand, they indirectly shape the immunosuppressive microenvironment by influencing immune cell differentiation, functional states, and metabolic reprogramming (15, 19, 20). Therefore, RNA epitranscriptomic regulation has become an important entry point for understanding tumor immune evasion.

In this review, we propose that RNA epitranscriptomic modifications regulate tumor immune evasion through an integrated post-transcriptional network rather than isolated gene-specific events. This network connects tumor-intrinsic immune visibility, immune checkpoint and inflammatory signaling, immune-cell remodeling, and metabolic adaptation of the tumor microenvironment. By organizing current evidence around these mechanistic layers, this review aims to clarify how RNA modifications collectively shape tumor immune escape and therapeutic response.

## 2 Overview of RNA epitranscriptomics

### 2.1 Definition and biological significance

RNA epitranscriptomics refers to the study of chemical modifications on RNA molecules that regulate RNA fate without altering the primary RNA sequence (3). These modifications are widely distributed in messenger RNA (mRNA), transfer RNA (tRNA), ribosomal RNA (rRNA), small nuclear RNA, long non-coding RNA, circular RNA, and other RNA species (21–23). Similar to DNA and histone epigenetic modifications, RNA modifications provide an additional regulatory layer for gene expression (24). However, RNA epitranscriptomic regulation mainly acts at the post-transcriptional level (24). RNA modifications influence multiple aspects of RNA metabolism, including RNA stability, splicing, nuclear export, localization, translation efficiency, degradation, and innate immune recognition (17, 24–27). These effects are usually mediated by specific regulatory proteins, including methyltransferases or acetyltransferases known as “writers,” demethylases or deacetylation-related enzymes known as “erasers,” and RNA-binding proteins known as “readers (28–30).” Through these regulators, RNA modifications dynamically control transcript fate and cellular responses. In cancer, dysregulated RNA epitranscriptomic modifications contribute to tumor initiation, progression, metastasis, metabolic adaptation, therapeutic resistance, and immune escape (30). Importantly, their biological effects are highly context-dependent. The same RNA modification may promote or suppress tumor progression depending on cancer type, cellular state, target transcript, reader protein, and tumor microenvironmental context (31–34). Therefore, understanding RNA epitranscriptomics provides important insight into how post-transcriptional regulation shapes cancer biology and tumor immunity.

### 2.2 Major RNA modifications

m^6^A is the most abundant internal modification in eukaryotic mRNA. It mainly occurs within the conserved RRACH motif, where R represents A or G and H represents A, C, or U. m^6^A is installed by methyltransferase complexes, including METTL3, METTL14, and WTAP, and can be removed by demethylases such as FTO and ALKBH5 (35–37). Its biological functions are mediated by reader proteins, especially YTH domain-containing proteins, IGF2BP family proteins, and other RNA-binding proteins (37). m^6^A regulates mRNA stability, splicing, nuclear export, translation, and degradation (38, 39). For example, YTHDF2 usually promotes mRNA decay, whereas YTHDF1 enhances translation efficiency (40, 41). In cancer, m^6^A is widely involved in cell proliferation, differentiation, stress responses, stemness maintenance, drug resistance, and immune regulation (42).

m^5^C is a methylation modification occurring at the fifth carbon of cytosine. It is widely present in tRNA, rRNA, mRNA, and non-coding RNAs (43, 44). m^5^C is mainly installed by NSUN family proteins, particularly NSUN2, as well as DNMT2 (45). Known m^5^C-associated reader proteins include YBX1 and ALYREF (46). Functionally, m^5^C regulates RNA export, stability, translation, and stress responses (47, 48). In mRNA, m^5^C can promote nuclear export and enhance transcript stability or translation efficiency (44).

ac^4^C is an RNA acetylation modification catalyzed mainly by NAT10 (49). It has been detected in mRNA, tRNA, and rRNA (49). Unlike m^6^A, which may either promote RNA decay or translation depending on reader proteins, ac^4^C is generally associated with increased transcript stability and enhanced translation efficiency (49, 50). By increasing transcript output, ac^4^C may support stress adaptation, metabolic reprogramming, and tumor progression (51, 52).

m¹A is a methylation mark at the N1 position of adenosine (53). It is mainly found in tRNA, rRNA, mitochondrial RNA, and the 5′ untranslated region of some mRNAs (53). Because m¹A carries a positive charge under physiological conditions, it can alter RNA secondary structure and affect translation initiation (54). m¹A is installed by methyltransferases such as TRMT6/TRMT61A and can be removed by demethylases such as ALKBH3 (55, 56). Functionally, m¹A participates in protein translation, stress responses, mitochondrial function, and metabolic regulation (56).

m^7^G was first identified as a key modification in the 5′ cap structure of mRNA, where it is essential for mRNA stability, nuclear export, and translation initiation (57). More recent studies have shown that m^7^G also occurs in tRNA, rRNA, and internal regions of mRNA (10, 58). Internal m^7^G modification is mainly catalyzed by the METTL1/WDR4 complex (59). m^7^G contributes to RNA structural stability, translation efficiency, and stress responses (60).

Ψ is one of the most abundant RNA modifications and is generated by the isomerization of uridine (61). It is widely present in tRNA, rRNA, small nuclear RNA, and mRNA (11, 61). Pseudouridylation is mainly catalyzed by pseudouridine synthases. Functionally, Ψ can enhance RNA structural stability, improve translation efficiency, and influence RNA-protein interactions (11, 62). Importantly, pseudouridine can reduce RNA immunogenicity and modulate innate immune recognition (63). This feature is especially relevant to mRNA-based therapeutics and vaccines.

m^6^Am is a cap-adjacent RNA modification located at the first transcribed nucleotide next to the mRNA 5′cap (64). It is catalyzed by PCIF1 and can be removed by FTO (65). m^6^Am is involved in regulating mRNA stability, translation, and transcript turnover (66). Because m^6^Am is located close to the mRNA cap structure, it may influence cap-dependent translation and mRNA decay (65). Nm refers to methylation at the 2′hydroxyl group of the ribose moiety. Nm is widely found in rRNA, tRNA, small nuclear RNA, and mRNA (67). It is involved in RNA stability, ribosome function, and translational control (68). Nm also plays an important role in distinguishing self from non-self RNA during innate immune recognition (68). By reducing recognition by innate immune sensors, Nm can dampen antiviral and inflammatory responses (68, 69).

A-to-I RNA editing is catalyzed by adenosine deaminases acting on RNA, especially ADAR family enzymes (14). During this process, adenosine is converted to inosine, which is usually interpreted as guanosine during translation or sequencing (70, 71). A-to-I editing can alter RNA sequence, RNA structure, splicing, stability, and protein coding potential (72, 73). It is particularly important in regulating double-stranded RNA sensing and interferon responses (74). To provide a concise overview of the major RNA epitranscriptomic modifications and their regulatory proteins, we summarized representative writers, erasers, readers, biological functions, and current limitations in **Table 1**. Overall, these emerging RNA modifications expand the scope of RNA epitranscriptomic regulation beyond classical methylation and acetylation marks. They are particularly relevant to innate immune sensing, RNA immunogenicity, interferon signaling, and therapeutic RNA design. Although their roles in tumor immune evasion are still being defined, they provide important directions for future research.

**Table 1:** Major RNA epitranscriptomic modifications and their regulatory proteins.

RNA modification	Main writers	Known erasers	Representative readers/effectors	Major biological effects	Current limitations	Ref
m^6^A	METTL3, METTL14, WTAP	FTO, ALKBH5	YTHDF1, YTHDF2, YTHDC1/2, IGF2BP1/2/3	Regulates RNA stability, decay, splicing, export, and translation. YTHDF1 promotes translation; YTHDF2 promotes RNA decay; IGF2BPs enhance mRNA stability.	Functional outcome is highly reader- and context-dependent.	(75, 78, 183–185)
m^5^C	NSUN family proteins, DNMT2	Less clearly defined	YBX1, ALYREF,YBX2, SRSF2	Regulates RNA stability, nuclear export, translation, and stress adaptation. ALYREF promotes mRNA export; YBX1 enhances RNA stability.	Reversibility and eraser enzymes remain incompletely defined.	(186–188)
ac^4^C	NAT10	Not clearly defined	NA	Enhances mRNA stability and translation efficiency; supports stress responses, metabolic adaptation, and tumor progression.	Reader proteins and eraser mechanisms remain poorly characterized.	(189, 190)
m^7^G	METTL1/WDR4; mRNA cap methyltransferase complexes	Not clearly defined	NA	Maintains mRNA cap function, RNA structural stability, translation initiation, and translational control. Internal m^7^G may regulate tRNA/mRNA function.	Internal m^7^G readers and reversibility remain insufficiently understood.	(10, 191)
m¹A	TRMT6/TRMT61A	ALKBH3; possibly ALKBH1 in some contexts	NA	Alters RNA structure, regulates translation initiation, mitochondrial RNA metabolism, and stress responses.	Reader proteins and context-specific functions require further clarification.	(192, 193)
m^6^Am	PCIF1	FTO	NA	Regulates cap-adjacent mRNA stability, transcript turnover, and possibly translation.	Functional distinction between m^6^A and m^6^Am remains under investigation.	(65, 66)
Pseudouridine/Ψ	Pseudouridine synthases	Not clearly defined	NA	Enhances RNA structural stability, translation efficiency, and reduces RNA immunogenicity.	Roles in tumor immunity remain less well defined.	(61, 194)
2′-O-methylation/Nm	Fibrillarin, CMTRs and other methyltransferases	Not clearly defined	NA	Regulates RNA stability, ribosome function, translation, and innate immune recognition.	Dynamic regulation and cancer-related functions remain incompletely characterized.	(13, 195)
A-to-I RNA editing	ADAR family enzymes	Generally irreversible	NA	Alters RNA sequence, structure, splicing, coding potential, and innate immune sensing; regulates dsRNA recognition and interferon responses.	Not a reversible methylation-like modification; context-specific immune effects require further study.	(14, 70)

## 3 Mechanistic principles of RNA epitranscriptomic regulation

The central mechanism by which RNA epitranscriptomic modifications regulate tumor immunity is the control of immune-related transcript fate. By modulating RNA stability, translation, splicing, export, and innate immune sensing, RNA modifications alter the abundance and function of transcripts involved in antigen presentation, checkpoint signaling, cytokine production, immune-cell recruitment, and metabolic remodeling.

### 3.1 RNA Stability and degradation

RNA modifications strongly influence transcript stability and degradation, thereby determining the abundance of specific mRNAs in cells (75, 76). Among these modifications, m^6^A is the best-characterized regulator of RNA decay. The m^6^A reader YTHDF2 recognizes m^6^A-modified transcripts and promotes their degradation by recruiting RNA decay machinery (77). Through this mechanism, m^6^A can reduce the expression of specific target genes involved in cell differentiation, stress responses, or immune regulation. However, m^6^A does not always promote RNA decay. In some contexts, m^6^A-modified transcripts are recognized by IGF2BP family proteins, including IGF2BP1, IGF2BP2, and IGF2BP3 (78). These readers enhance mRNA stability and increase target transcript abundance (78). Therefore, the effect of m^6^A on RNA stability depends largely on which reader protein is recruited. Other RNA modifications also regulate RNA stability. m^5^C, installed mainly by NSUN2, can stabilize target transcripts through reader proteins such as YBX1 (45). This mechanism may help maintain the expression of genes involved in tumor cell survival, migration, and stress adaptation (46, 79). ac^4^C, catalyzed by NAT10, generally enhances mRNA stability and increases transcript output (52). In tumor cells, NAT10-mediated ac^4^C modification may stabilize mRNAs involved in proliferation, metabolism, and adaptation to stress (80). Together, these examples show that RNA modifications regulate gene expression not only by turning transcripts “on” or “off,” but by fine-tuning transcript lifespan. This mechanism is particularly important in cancer, where stabilization of oncogenic or immune-suppressive transcripts can promote tumor progression and immune evasion.

### 3.2 Translation efficiency

RNA epitranscriptomic modifications also regulate how efficiently mRNAs are translated into proteins. This regulation allows cells to rapidly adjust protein production without changing mRNA abundance. m^6^A can promote translation through reader proteins such as YTHDF1 (81). YTHDF1 recognizes m^6^A-modified transcripts and enhances their translation efficiency by facilitating ribosome loading or interaction with translation initiation machinery (81). This mechanism can increase the production of proteins involved in cell proliferation, stress adaptation, immune checkpoint regulation, or antigen presentation. In contrast, when m^6^A-modified transcripts are bound by YTHDF2, they may be degraded rather than translated, again emphasizing the reader-dependent nature of m^6^A function (82, 83). m^7^G is another important modification linked to translation. The classical m^7^G cap at the 5′ end of mRNA is essential for cap-dependent translation initiation (84). In addition, internal m^7^G modification, mainly installed by the METTL1/WDR4 complex, can regulate translation by affecting RNA structure and codon decoding, especially in tRNAs (59). Overall, RNA modifications regulate translation by affecting ribosome recruitment, RNA structure, tRNA function, cap-dependent translation, and transcript accessibility. In cancer, this provides a rapid mechanism for increasing the production of proteins that support malignant growth, therapy resistance, and immune escape.

### 3.3 RNA splicing, export, and localization

Beyond RNA stability and translation, RNA modifications also regulate RNA processing, nuclear export, and subcellular localization (85, 86). These processes determine where and when transcripts become available for translation or immune recognition. m^6^A plays an important role in pre-mRNA splicing and nuclear RNA processing. The nuclear m^6^A reader YTHDC1 can bind m^6^A-modified pre-mRNAs and influence alternative splicing by interacting with splicing factors (85). Through this mechanism, m^6^A may generate different transcript isoforms with distinct biological functions. m^5^C is closely related to RNA export. The m^5^C reader ALYREF recognizes m^5^C-modified mRNAs and promotes their export from the nucleus to the cytoplasm (87). This allows selected transcripts to be efficiently transported for translation.

## 4 Context dependency and functional plasticity of RNA modification regulators

### 4.1 Oncogenic functions of RNA epitranscriptomics in cancer

Accumulating evidence indicates that RNA epitranscriptomic regulators not only participate in tumor cell proliferation and survival, but also promote oncogenesis by modulating DNA damage repair, therapeutic tolerance, metabolic reprogramming, and the immunosuppressive tumor microenvironment (34, 76). Importantly, these effects are not determined by a single RNA modification or an individual target gene. Instead, they are mediated through a continuous regulatory axis involving modification enzymes, RNA marks, reader proteins, and functional target transcripts. In the context of therapy resistance, m^6^A regulators can enhance DNA damage repair and thereby support tumor cell survival under chemotherapeutic pressure. For example, in triple-negative breast cancer, cisplatin treatment induces increased m^6^A modification. Mechanistically, HDAC2 mediates METTL3 delactylation, which strengthens the interaction between METTL3 and WTAP and subsequently increases m^6^A modification on DNA damage repair-related transcripts (88). This promotes tumor cell survival during cisplatin treatment. This study suggests that the oncogenic function of METTL3 is not solely dependent on its methyltransferase activity, but is also regulated by post-translational modifications such as lactylation and delactylation, highlighting the complex crosstalk among metabolic state, epigenetic regulation, and RNA modification (88). Similarly, the m^6^A reader YTHDF1 promotes breast cancer progression by enhancing DNA replication and DNA damage repair. YTHDF1 regulates *E2F8* mRNA stability in a METTL14-dependent manner, thereby facilitating S-phase entry, DNA replication, and DNA repair. Consequently, YTHDF1 increases tumor cell resistance to doxorubicin, cisplatin, and the PARP inhibitor olaparib (89). These findings indicate that m^6^A reader proteins are not merely passive interpreters of RNA modification signals, but can serve as functional effectors linking RNA modification to therapeutic resistance (89). Beyond chemoresistance, RNA modifications may also shape an immunosuppressive microenvironment through metabolic reprogramming. A representative example is ac^4^C modification. In cervical cancer, elevated NAT10 expression is associated with poor prognosis. HOXC8 activates NAT10 transcription, which enhances ac^4^C modification of FOXP1 mRNA and increases its translation efficiency (90). *FOXP1* subsequently upregulates *GLUT4* and *KHK* expression, thereby promoting glycolysis and lactate secretion. The lactate-enriched tumor microenvironment further strengthens the immunosuppressive function of tumor-infiltrating Tregs and weakens antitumor immunity (90). Notably, NAT10 knockdown enhances PD-L1 blockade-mediated tumor regression *in vivo*, suggesting that the NAT10/ac^4^C axis not only supports metabolic adaptation but may also serve as a potential target to improve immunotherapy responses (90). This finding is conceptually important because it demonstrates that the oncogenic function of RNA modification regulators is not restricted to tumor-intrinsic proliferation, but can also remodel the tumor immune microenvironment through metabolic products such as lactate. m^5^C modification also contributes to therapeutic resistance and tumor recurrence. In the context of EGFR-TKI treatment, RNA m^5^C hypermethylation and increased NSUN2 expression are closely associated with intrinsic resistance to EGFR-TKIs. NSUN2 methylates the coding sequence region of *QSOX1* and promotes YBX1-dependent enhancement of *QSOX1* translation (46), leading to gefitinib resistance and tumor recurrence. Conversely, genetic inhibition of NSUN2 induces tumor regression and overcomes intrinsic gefitinib resistance. These results suggest that m^5^C modification not only regulates RNA stability and export, but can also promote targeted therapy failure by increasing the translation of resistance-associated transcripts (46). However, several limitations should be noted. Many current studies remain focused on a single cancer type, a single RNA modification, or a limited number of target genes. Whether different RNA modifications act cooperatively, competitively, or compensatorily remains insufficiently understood. In addition, although some studies have established associations between RNA modification regulators and drug resistance or immunosuppression, their cell-type-specific functions require further clarification. The same RNA-modifying enzyme may exert distinct or even opposite effects in tumor cells and immune cells. Therefore, future studies should integrate RNA modification mapping, reader-specific functional assays, single-cell omics, and immune functional analyses to better define the causal roles of RNA modification regulators in tumor progression and immune escape.

### 4.2 Tumor-suppressive functions: context-dependent protective roles of RNA modification regulators

Although RNA epitranscriptomic modifications are often considered drivers of tumor progression and immune evasion, their functions are not uniformly oncogenic (91). In specific cancer types, cellular states, and target transcript contexts, RNA modification regulators may also exert tumor-suppressive effects by promoting the degradation of oncogenic transcripts, suppressing tumor stemness, enhancing cell death sensitivity, and limiting metastatic signaling (92, 93). Therefore, RNA modification regulators should not be simply classified as oncogenes or tumor suppressors. Instead, their functions should be interpreted according to the specific writer/eraser/reader, target RNA, and cellular context. The m^6^A writer METTL14 is a representative tumor-suppressive regulator. In colorectal cancer, reduced METTL14 expression is associated with poor prognosis. Mechanistically, METTL14 mediates m^6^A modification of *SOX4* mRNA and promotes YTHDF2-dependent *SOX4* mRNA degradation, thereby inhibiting EMT, PI3K/Akt signaling, tumor migration (94), and metastasis. This finding indicates that m^6^A does not always enhance oncogenic gene expression. In certain reader-dependent contexts, m^6^A can also promote the degradation of oncogenic transcripts and thereby exert tumor-suppressive effects (94). Moreover, METTL14 itself is regulated by upstream RNA regulatory networks. For instance, SLC27A5 and PABPC1 can modulate alternative splicing and polyadenylation of METTL14 mRNA, increase METTL14 expression, and suppress liver cancer stem cell properties, further suggesting that the tumor-suppressive activity of METTL14 is controlled by multilayered regulatory mechanisms. In triple-negative breast cancer, loss of METTL14 reduces the global m^6^A level and allows *YAP1* mRNA to escape YTHDF2-mediated degradation, leading to activation of Hippo-independent YAP1 signaling and maintenance of tumor stemness (95). This study suggests that tumor cells may stabilize stemness-promoting transcripts by epigenetically silencing METTL14. Therefore, restoration of the METTL14-m^6^A-YTHDF2 axis may help suppress TNBC stemness (95). In addition to writers, m^6^A readers may also exert tumor-suppressive functions. In lung cancer, YTHDC1 regulates *FSP1* mRNA alternative polyadenylation and stability, thereby reducing FSP1 protein expression, enhancing ferroptosis sensitivity, and limiting tumor progression (96). Conversely, YTHDC1 downregulation stabilizes *FSP1* mRNA, increases ferroptosis resistance, and promotes lung cancer progression. This finding suggests that reader proteins not only recognize RNA modifications but also regulate RNA isoforms and cell death pathways to exert tumor-suppressive effects (96). Notably, FTO, an m^6^A eraser commonly considered oncogenic, may also function as a tumor suppressor in certain contexts. In ovarian cancer, FTO expression is reduced in tumors and cancer stem cells. FTO removes m^6^A marks from the 3′UTRs of *PDE1C* and *PDE4B* mRNAs, decreases their stability, enhances cAMP signaling, and suppresses ovarian cancer stem cell self-renewal and tumor formation (97). This finding highlights the cancer type-dependent function of FTO and cautions against defining it simply as an oncogenic factor (97). Nevertheless, current evidence regarding tumor-suppressive RNA modification pathways remains largely centered on individual regulatory axes. How these mechanisms influence the tumor immune microenvironment has not been systematically defined. Future studies integrating RNA modification mapping, cell-type-specific models, and immune functional assays will be necessary to clarify the causal relationship between tumor-suppressive RNA epitranscriptomic regulation and antitumor immunity.

## 5 Molecular mechanisms of RNA epitranscriptomics in tumor immune escape

**Figure 1:**
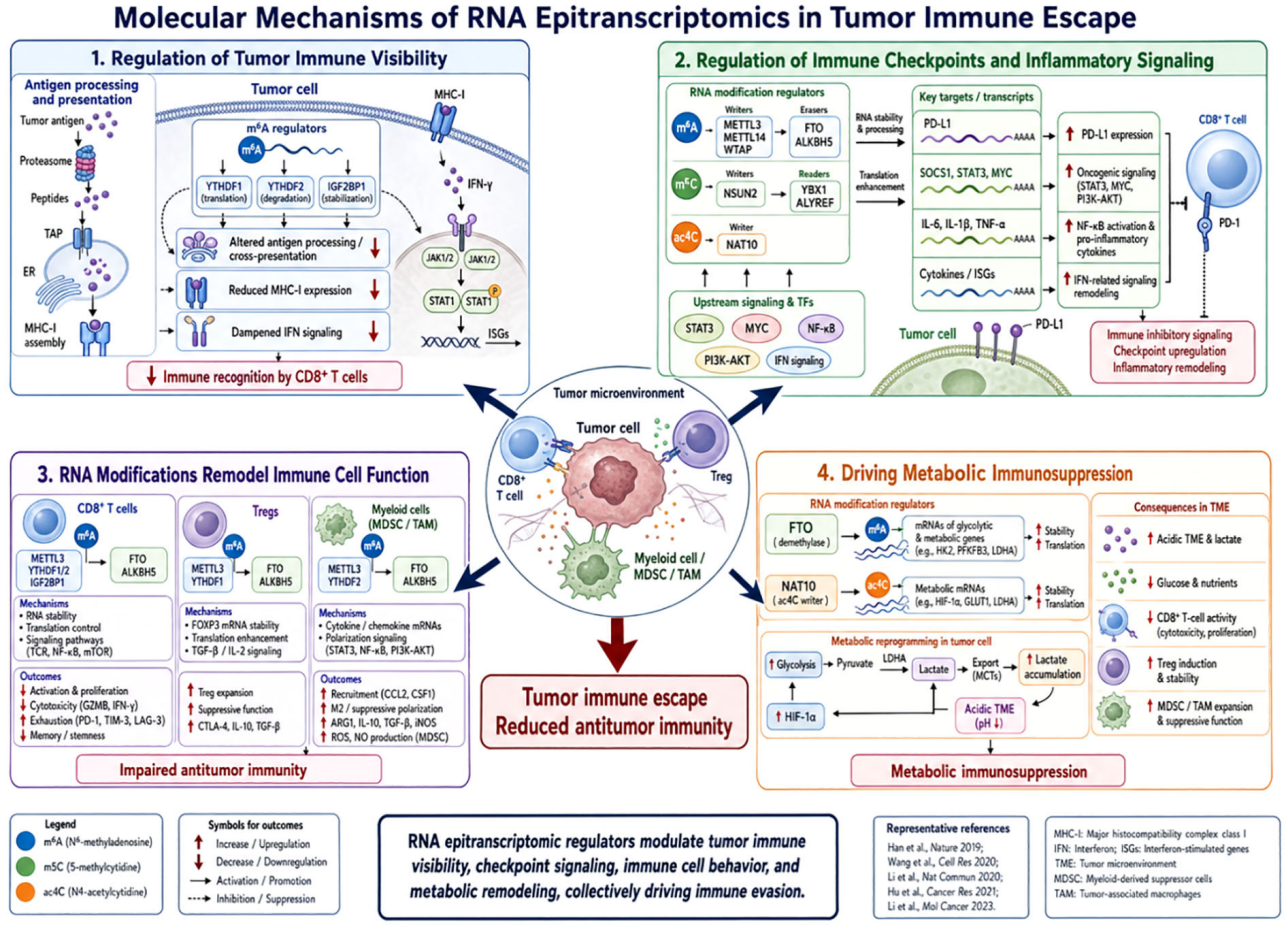
Molecular mechanisms of RNA epitranscriptomics in tumor immune escape. RNA epitranscriptomic regulators promote tumor immune evasion through multiple interconnected mechanisms. First, m^6^A regulators, including YTHDF1, YTHDF2, and IGF2BP1, modulate antigen processing, MHC-I expression, and IFN–JAK–STAT signaling, thereby reducing tumor immune visibility and weakening CD8^+^ T-cell recognition. Second, RNA modifications regulate immune checkpoint and inflammatory signaling. m^6^A writers such as METTL3, METTL14, and WTAP, erasers such as FTO and ALKBH5, as well as m^5^C- and ac^4^C-related regulators including NSUN2, YBX1/ALYREF, and NAT10, affect the stability, processing, or translation of transcripts related to PD-L1, cytokines, and oncogenic inflammatory pathways such as STAT3, MYC, NF-κB, PI3K–AKT, and IFN-related signaling. Third, RNA modifications reshape immune cell function by regulating CD8^+^ T-cell activation and exhaustion, promoting Treg expansion and suppressive activity, and enhancing the recruitment or immunosuppressive polarization of myeloid cells, including MDSCs and TAMs. Fourth, RNA epitranscriptomic regulators drive metabolic immunosuppression by controlling glycolytic and metabolic transcripts, increasing lactate production, acidifying the tumor microenvironment, limiting CD8^+^ T-cell activity, and supporting Treg and myeloid suppressor cell accumulation. Together, these mechanisms converge to remodel tumor immune visibility, checkpoint signaling, immune cell behavior, and metabolic states, ultimately promoting tumor immune escape and reducing antitumor immunity.

These mechanisms are highly interconnected. Regulation of immune checkpoint molecules can cooperate with metabolic remodeling, inflammatory signaling, and immune-cell dysfunction to establish an immunosuppressive tumor microenvironment. Thus, RNA epitranscriptomic regulation acts as a molecular bridge linking tumor-intrinsic gene expression programs with extrinsic immune suppression. The major molecular mechanisms by which RNA epitranscriptomic regulation promotes tumor immune escape are summarized in **Figure 1**. The key RNA epitranscriptomic regulators involved in tumor immune escape and antitumor immune regulation are summarized in **Table 2**.

**Table 2:** RNA epitranscriptomic regulators involved in tumor immune escape and antitumor immune regulation.

RNA regulator	Immune regulatory function	Main target genes	Molecular mechanism	References
YTHDF1	Suppresses antigen presentation and CD8^+^ T-cell activation; regulates DC maturation and T-cell infiltration	Lysosomal proteases; MHC-I antigen presentation pathway; IFN-γ signaling components	Promotes translation of lysosomal proteases, accelerates antigen degradation, weakens MHC-I-mediated antigen presentation and DC cross-presentation; context-dependently enhances IFN-γ signaling and DC maturation	(100, 101)
YTHDF2	Promotes antigen escape; suppresses DC cross-presentation and CD8^+^ T-cell activation	Immune-related transcripts; Notch pathway components; MHC-I cross-presentation-related molecules	Acts as an m^6^A reader to promote degradation of immune-related transcripts; degrades Notch pathway components and impairs DC-mediated cross-presentation	(102, 106)
lnc-Dpf3	Indirectly regulates antigen presentation and immune priming	HIF-1α glycolysis pathway; DC migration-related programs	m^6^A-regulated lncRNA controls metabolic reprogramming and affects DC migration, thereby modulating antigen presentation	(103)
circCRIM1	Enhances CD8^+^ T-cell and NK-cell effector functions	IGF2BP1; HLA-related molecules	Competitively binds IGF2BP1 and modulates the stability of HLA-related molecules, improving antitumor immune recognition	(196)
IGF2BP1	Promotes immune-cold phenotype; suppresses antigen presentation and immune infiltration	IRF1; MHC-I; IFN-γ signaling pathway	Promotes IRF1 degradation, suppresses IFN-γ signaling, reduces MHC-I expression, and limits immune cell infiltration	(104)
METTL3	Enhances PD-L1-mediated immune suppression; regulates innate immune activation and TIME remodeling	PD-L1 mRNA; JAK1; TLR4; endogenous retroelements	Deposits m^6^A marks on PD-L1 mRNA and enhances stability through IGF2BP proteins; promotes JAK1 translation and activates JAK1–STAT3 signaling; enhances TLR4 translation and activates TLR4–MyD88–NF-κB signaling; METTL3 methylation at K513 strengthens enzymatic activity and suppresses type I IFN responses	(91, 109, 197)
METTL14	Regulates immune suppression and CD8^+^ T-cell exhaustion	PD-L1 mRNA; EBI3	Installs m^6^A marks on PD-L1 mRNA to enhance checkpoint signaling; in TAMs, METTL14-dependent m^6^A regulation induces CD8^+^ T-cell exhaustion through EBI3	(91, 109, 197)
WTAP	Promotes immune checkpoint regulation and metabolic immune suppression	PD-L1 mRNA; piR-1170-related pathway; TCR signaling transcripts	Participates in m^6^A deposition on PD-L1 mRNA; piR-1170 regulates WTAP-mediated m^6^A modification to coordinate metabolic reprogramming with PD-L1-dependent immune suppression; controls stability of TCR signaling-related transcripts	(91, 109, 136, 197)
IGF2BP1/IGF2BP2	Enhances immune checkpoint signaling	PD-L1; c-Myc	Stabilizes PD-L1 mRNA by binding m^6^A sites and indirectly promotes PD-L1 transcription through c-Myc	(198, 199)
ALKBH5	Context-dependent regulation of PD-L1 and immune suppression; promotes TAM recruitment under hypoxia	PD-L1 mRNA; NEAT1; CXCL8	Removes m^6^A marks from PD-L1 mRNA and regulates its stability/degradation; hypoxia-induced ALKBH5 stabilizes NEAT1, promotes paraspeckle formation, relieves CXCL8 repression, and enhances TAM recruitment	(149, 177, 200)
NAT10	Promotes PD-L1 transcription and immune suppression; may also enhance immune activation through lncRNA regulation	ETS2; HDAC4; NF-κB; lncRNA-GAS5	Mediates ac^4^C modification to stabilize ETS2 or HDAC4, activating PD-L1 transcription or the NAT10–HDAC4–NF-κB feedback axis; ac^4^C-regulated lncRNA-GAS5 activates the p53–IRF1 axis and type I IFN signaling	(115, 177, 201)
NSUN2	Suppresses CD8^+^ T-cell infiltration and activity; promotes metabolic immune evasion	PD-L1 mRNA; ALYREF; SOAT2	Mediates m^5^C modification and stabilizes PD-L1 mRNA through the NSUN2–ALYREF axis; regulates metabolic genes such as SOAT2 to reprogram tumor metabolism and suppress CD8^+^ T-cell function	(131, 178)
YTHDF3	Context-dependent suppression of PD-L1 expression	PD-L1-related target mRNAs	Promotes degradation of target mRNAs and suppresses PD-L1 expression	(114)
circRHBDD1	Enhances PD-L1 stability and immune checkpoint signaling	IGF2BP2; PD-L1	Inhibits IGF2BP2 degradation, thereby stabilizing PD-L1 and strengthening immune evasion	(108)
circIGF2BP3	Promotes PD-L1 protein stability	PKP3; OTUB1; PD-L1	Promotes PD-L1 deubiquitination through the PKP3–OTUB1 axis, increasing PD-L1 protein stability	(108)
piR-1170	Coordinates metabolic reprogramming and PD-L1-mediated immune suppression	WTAP; PD-L1-related m^6^A pathway	Regulates WTAP-mediated m^6^A modification and links tumor metabolism with checkpoint-dependent immune evasion	(119)
METTL3–YTHDF1 axis	Promotes immunosuppressive myeloid cell function	JAK1; STAT3 pathway	METTL3 deposits m^6^A on JAK1 mRNA; YTHDF1 enhances JAK1 translation, activating JAK1–STAT3 signaling in tumor-infiltrating myeloid cells	(200)
YTHDF1–MCT1 axis	Suppresses CD8^+^ T-cell cytotoxicity through lactate metabolism	MCT1	Stabilizes or enhances expression of the lactate transporter MCT1, leading to lactate accumulation and reduced CD8^+^ T-cell cytotoxicity	(202)
METTL3–BHLHE41–CXCL1 axis	Promotes MDSC recruitment and immunosuppressive TIME formation	BHLHE41; CXCL1	Enhances CXCL1-related chemokine signaling, promoting MDSC migration into the tumor microenvironment	(130)
YTHDF1–p65–CXCL1 axis	Promotes MDSC recruitment	p65; CXCL1; CXCR2	Enhances translation of p65, increases CXCL1 expression, and recruits MDSCs through the CXCL1–CXCR2 axis	(81)
YTHDF2–NF-κB axis	Promotes MDSC expansion and radioresistance-related immune suppression	NF-κB pathway; MDSC-related inflammatory signals	Forms a positive feedback loop with NF-κB signaling under radiotherapy, enhancing MDSC expansion and suppressive function	(81)
YTHDF2–ETV5 axis	Coordinates angiogenesis and immune escape	ETV5; VEGFA; PD-L1	Promotes translation of ETV5, leading to increased VEGFA and PD-L1 expression	(149)
METTL1	Promotes glycolysis-associated immune suppression	PKM2; CD155	Mediates m^7^G modification, enhances PKM2 expression, promotes glycolysis, and induces immunosuppressive signals such as CD155	(148)
RBM15	Enhances PD-L1 expression and immune suppression	m^6^A-modified circRNAs; JAK2–STAT3–STAT5 pathway; PD-L1	Promotes m^6^A modification of circular RNAs and activates JAK2–STAT3–STAT5 signaling, leading to increased PD-L1 expression	(107)
FTO	Promotes glycolytic immune suppression; regulates innate immune escape and T-cell responses	c-Jun; LILRB4; Th1-related transcripts	Removes m^6^A marks from target transcripts, upregulates c-Jun-driven glycolysis, suppresses CD8^+^ T-cell function, and increases LILRB4-mediated immune evasion; FTO inhibition increases m^6^A and enhances immune activation	(135, 143, 203, 204)
METTL3–TLR4 axis	Activates innate immune signaling and inflammatory cytokine release	TLR4; MyD88; NF-κB	m^6^A modification enhances TLR4 mRNA translation and delays degradation, activating TLR4–MyD88–NF-κB signaling	(121)
METTL3-mediated dsRNA regulation	Enhances RLR signaling and antitumor immunity	Endogenous double-stranded RNA; A-to-I RNA editing; RLR pathway	Suppresses A-to-I RNA editing, promotes endogenous dsRNA accumulation, and activates RLR signaling	(205)
YTHDF2–RIG-I axis	Suppresses innate immune sensing and CD8^+^ T-cell infiltration	RIG-I mRNA; RLR signaling pathway	Promotes degradation of RIG-I-encoding mRNA, thereby inhibiting RLR signaling and reducing antitumor immune infiltration	(206)
Pseudouridine-related regulators	Reduce RNA immunogenicity and suppress innate/T-cell-mediated antitumor immunity	Endogenous dsRNA; TLR3/7/8-related recognition pathways	Pseudouridine reduces endogenous dsRNA accumulation and decreases activation of innate immune sensors; inhibition enhances innate immune activation and T-cell-mediated antitumor responses	(204, 207)
METTL3 in T cells	Regulates T-cell differentiation, proliferation, and homeostasis	TCF7; SOCS family mRNAs; IL-7–STAT5 pathway	Stabilizes transcription factor mRNAs such as TCF7 to promote follicular helper T-cell differentiation; regulates SOCS mRNA degradation to control IL-7–STAT5 signaling	(132, 133)
m^5^C-related regulation	Promotes Th17 differentiation	IL-17 and inflammatory transcripts	Stabilizes inflammatory transcripts, including IL-17, thereby promoting Th17 differentiation	(134)
PDCD1 m^6^A regulation	Controls T-cell exhaustion and immunotherapy response	PDCD1 mRNA	m^6^A-dependent regulation of PDCD1 mRNA affects T-cell exhaustion and response to immune checkpoint therapy	(208)

### 5.1 Regulation of tumor immune visibility

Epitranscriptomic RNA modifications regulate tumor immune visibility by modulating antigen processing and presentation, MHC-I expression, IFN signaling, and dendritic cell-mediated cross-presentation (81, 98, 99). These processes determine whether tumor cells can be effectively recognized and eliminated by CD8^+^ T cells. Current evidence supports a central concept that RNA modification-mediated post-transcriptional regulation links tumor-intrinsic gene expression programs to immune escape by altering the stability, translation, and degradation of immune-related transcripts (100, 101). m^6^A readers play particularly important roles in this process. YTHDF1 promotes the translation of lysosomal proteases, accelerates antigen degradation, and consequently weakens MHC-I-mediated antigen presentation and dendritic cell cross-presentation. This suppresses CD8^+^ T-cell activation and contributes to immune evasion (100). This mechanism suggests that RNA modifications do not necessarily reduce antigen expression directly; instead, they may impair immune recognition by enhancing antigen degradation. YTHDF2 also regulates tumor immune visibility, but its function appears more context-dependent. As an m^6^A reader, YTHDF2 can promote the degradation of immune-related transcripts, reduce antigen expression, and facilitate antigen escape (102) In B-cell malignancies and radiotherapy-treated settings, YTHDF2 degrades key components of the Notch pathway, suppresses dendritic cell-mediated MHC-I cross-presentation, and weakens CD8^+^ T-cell activation, thereby promoting immune escape and metastasis (102).

RNA modifications may also regulate immune visibility indirectly through non-coding RNAs and RNA-binding protein networks. For example, m^6^A-regulated *lncRNA Dpf3* affects metabolic reprogramming through the HIF-1α glycolytic pathway and regulates dendritic cell migration, thereby influencing antigen presentation and immune priming (103). In addition, the m^6^A reader IGF2BP1 suppresses IFN-γ signaling by promoting IRF1 degradation, which reduces MHC-I expression, limits immune-cell infiltration, and contributes to an immune-cold phenotype (104). These findings indicate that RNA modifications regulate tumor immune visibility not only through antigen-processing machinery, but also through IFN signaling, metabolic state, and immune-cell trafficking.

Immune “hot” tumors generally refer to tumors with abundant immune-cell infiltration, active antigen presentation, interferon signaling, and pre-existing antitumor T-cell responses (105). In contrast, immune “cold” tumors are characterized by poor immune infiltration, defective antigen presentation, immunosuppressive stromal or myeloid compartments, and limited responsiveness to immune checkpoint blockade (105). In contrast, loss of YTHDF1 markedly enhances antigen presentation, converts immune cold tumors into immune hot tumors, and improves the response to immune checkpoint therapy (101). However, current studies also reveal considerable complexity and context dependency. YTHDF1 is generally considered to weaken antigen presentation and promote immune escape (100), yet in certain gastric cancer models, YTHDF1 has been reported to enhance IFN-γ signaling, promote dendritic cell maturation, increase T-cell infiltration, and restore antitumor immunity (101). Similarly, YTHDF2 can promote antigen escape by degrading immune-related transcripts in tumor cells (106), but it may also stabilize *CX3CL1* mRNA in hepatocytes, promote CD8^+^ T-cell recruitment, and enhance antitumor immunity (107). These apparently contradictory findings suggest that the function of a given reader protein depends on tumor type, cellular source, target transcript, and immune microenvironmental context, rather than on the RNA modification itself. Mechanistically, the regulation of antigen presentation, MHC-I expression, and IFN-related transcripts by YTHDF1, YTHDF2, and IGF2BP1 is supported by multiple studies. Nevertheless, several key questions remain unresolved. First, many studies focus on individual regulatory axes, such as YTHDF1–lysosomal proteases, YTHDF2–Notch signaling, or IGF2BP1–*IRF1*, but whether these pathways cooperate or compensate for each other within the same tumor context remains unclear. Second, some conclusions are still based primarily on expression correlations or model-based inference and require direct validation using RNA modification mapping, reader-specific rescue assays, and functional immune assays (105). Third, the same RNA modification regulator may exert distinct or even opposite effects in tumor cells and immune cells, highlighting the need for cell-type-specific models. Overall, RNA epitranscriptomic modifications shape tumor immune visibility by regulating antigen degradation, MHC-I expression, IFN signaling, dendritic cell cross-presentation, and CD8^+^ T-cell activation. The key insight is not that a single reader protein or target gene determines immune recognition, but that RNA modifications form a post-transcriptional regulatory network connecting RNA fate control with tumor immune surveillance. Future studies should move beyond single molecular axes and define cell-type-specific and network-level mechanisms to identify RNA modification pathways with real translational potential for improving immunotherapy responses.

### 5.2 Regulation of immune checkpoints and inflammatory signaling

Aberrant activation of immune checkpoint pathways, especially the PD-1/PD-L1 axis, is a major mechanism by which tumors suppress T-cell-mediated antitumor immunity. Increasing evidence indicates that RNA epitranscriptomic modifications regulate immune checkpoint expression and inflammatory signaling at multiple levels, including mRNA stability, translation, transcriptional activation, protein stability, and noncoding RNA-mediated regulatory networks (90, 100, 108). Thus, RNA modifications provide a post-transcriptional layer that connects oncogenic signaling, inflammatory pathways, and immune escape. Among these mechanisms, m^6^A modification is one of the best-characterized regulators of PD-L1 expression. m^6^A writers, including METTL3, METTL14, and WTAP, can deposit m^6^A marks in the 3′ untranslated region of *PD-L1* mRNA (91, 99, 109). These marks are recognized by IGF2BP family proteins, such as IGF2BP2 and IGF2BP3, which enhance *PD-L1* mRNA stability and increase *PD-L1* expression (110, 111). Functionally, this suppresses CD8^+^ T-cell cytotoxicity and promotes T-cell exhaustion. This writer-reader axis suggests that immune checkpoint expression is not only transcriptionally regulated, but also highly dependent on RNA modification-mediated mRNA stabilization (112). However, the regulation of *PD-L1* by RNA modifications is not unidirectional. RNA demethylases such as ALKBH5 dynamically regulate PD-L1 mRNA fate by removing m^6^A marks (113) In some contexts, loss of ALKBH5 promotes YTHDF2-dependent *PD-L1* mRNA degradation and enhances T-cell activity (113). However, in other tumor settings, ALKBH5 may help maintain *PD-L1* expression and promote immune suppression. Similarly, YTHDF3 has been reported to suppress *PD-L1* expression by promoting degradation of target mRNAs (114). These findings highlight that the effect of RNA modification regulators on immune checkpoint expression depends on tumor type, reader availability, target transcript context, and the immune microenvironment.

Other RNA modifications also converge on immune checkpoint regulation. NAT10-mediated ac^4^C modification can stabilize transcriptional regulators such as *ETS2 or HDAC4*, thereby activating *PD-L1* transcription or reinforcing positive feedback loops such as the NAT10–HDAC4–NF-κB axis (115). m^5^C modification also contributes to immune suppression. For example, NSUN2-mediated m^5^C modification can stabilize *PD-L1* mRNA through the ALYREF-dependent pathway and reduce CD8^+^ T-cell infiltration (116). These studies suggest that different RNA modifications may converge on the same immunosuppressive output, especially PD-L1 upregulation, through distinct molecular routes. RNA-binding proteins and noncoding RNAs further expand this regulatory network. IGF2BP1 and IGF2BP2 can stabilize *PD-L1* mRNA by recognizing m^6^A-modified regions and may also indirectly promote *PD-L1* transcription through c-Myc-related mechanisms (117, 118). Circular RNAs and piRNAs also regulate *PD-L1* expression by modulating RNA-binding protein stability or acting as competing endogenous RNAs. For example, circRHBDD1 enhances PD-L1 stability by preventing IGF2BP2 degradation, whereas circIGF2BP3 promotes PD-L1 deubiquitination through the PKP3–OTUB1 axis (108). piRNAs such as piR-1170 can regulate WTAP-mediated m^6^A modification and coordinate metabolic reprogramming with PD-L1-dependent immune suppression (119). These findings indicate that RNA modifications regulate immune checkpoints through multilayered RNA-protein and noncoding RNA networks rather than through a single linear pathway. In addition to immune checkpoints, RNA epitranscriptomic modifications regulate inflammatory and innate immune signaling. Pattern recognition receptor pathways, including TLR and RIG-I-like receptor signaling, are central to tumor immune surveillance (120). RNA modifications can either enhance or suppress these pathways by altering RNA immunogenicity and the expression of innate immune sensors. For instance, METTL3 can modify *TLR4* mRNA, enhance its translation, delay its degradation, and activate the TLR4–MyD88–NF-κB pathway, leading to neutrophil activation and cytokine release (121). Conversely, RNA modifications may also dampen innate immune recognition. Although this mechanism helps prevent inappropriate immune activation under physiological conditions, tumors may exploit it to reduce immune recognition (122). FTO also contributes to immune evasion by increasing immunosuppressive molecules such as LILRB4; pharmacological degradation or inhibition of FTO can increase m^6^A levels, reduce LILRB4 expression, and enhance immune activation (122).

Overall, RNA epitranscriptomic modifications regulate immune checkpoints and inflammatory signaling through several interconnected mechanisms: stabilizing *PD-L1* mRNA, regulating *PD-L1* transcription and protein stability, modulating NF-κB and JAK-STAT signaling, controlling PRR-mediated innate immune activation, and shaping noncoding RNA-mediated immune regulatory networks. A key insight is that these pathways are not independent. Immune checkpoint expression, inflammatory signaling, RNA immunogenicity, and metabolic reprogramming often reinforce one another to establish an immunosuppressive tumor microenvironment. Nevertheless, several issues remain unresolved.

### 5.3 RNA modifications remodel immune suppression cell function

RNA epitranscriptomic modifications remodel the tumor immune microenvironment by regulating the recruitment, differentiation, activation, and suppressive functions of multiple immune-cell populations, including CD8^+^ T cells, Tregs, TAMs, MDSCs, NK cells, and DCs (123–125). Rather than acting only within tumor cells, RNA modifications also shape the behavior of immune cells and establish intercellular regulatory circuits that promote either immune suppression or antitumor immunity (125, 126). Myeloid cells are major targets of RNA modification-mediated immune remodeling. m^6^A modification plays an important role in tumor-infiltrating myeloid cells, TAMs, and MDSCs (127). For example, METTL3 deposits m^6^A on *JAK1* mRNA and enhances its translation through YTHDF1, activating the JAK1–STAT3 pathway and promoting the immunosuppressive function of tumor-infiltrating myeloid cells (128). Importantly, tumor-derived lactate can further upregulate METTL3 through histone lactylation and enhance its RNA-binding activity, suggesting a direct link among metabolic reprogramming, epigenetic regulation, RNA modification, and immune suppression (128). Under hypoxia, ALKBH5 removes m^6^A marks from lncRNAs such as *NEAT1*, stabilizes these transcripts, promotes paraspeckle formation, and relieves repression of *CXCL8* transcription, thereby enhancing TAM recruitment and immune suppression (129). RNA modifications also regulate chemokine networks that recruit immunosuppressive myeloid cells. METTL3 enhances the BHLHE41–*CXCL1* axis to promote MDSC migration (130). YTHDF1 increases *CXCL1* expression by enhancing p65 translation, thereby recruiting MDSCs through the CXCL1–CXCR2 axis (81). Under radiotherapy, YTHDF2 can form a positive feedback loop with NF-κB signaling, promoting MDSC expansion and suppressive function (98). In addition, m^5^C modification mediated by NSUN2 regulates metabolic genes such as *SOAT2*, reprograms energy metabolism, and suppresses CD8^+^ T-cell activity (131). These findings consistently indicate that RNA modifications promote immune evasion not only by affecting tumor cells, but also by shaping myeloid-cell recruitment and suppressive phenotypes.

T cells are another critical layer of RNA modification-dependent immune regulation. m^6^A is essential for CD4^+^ T-cell differentiation and homeostasis. METTL3 promotes follicular helper T-cell differentiation by stabilizing transcripts encoding transcription factors such as *TCF7* (132). m^6^A also regulates the degradation of SOCS family mRNAs and controls IL-7–STAT5 signaling, thereby influencing T-cell homeostasis, proliferation, and differentiation (133). Other RNA modifications also participate in T-cell fate decisions. m^5^C promotes Th17 differentiation by stabilizing inflammatory transcripts such as IL-17 (134), whereas FTO-mediated demethylation supports Th1 expansion and IFN-γ production (135). WTAP regulates T-cell receptor signaling by controlling the stability of related transcripts, thereby affecting T-cell activation, apoptosis, and survival (136).

Within the tumor immune microenvironment, RNA modifications regulate T-cell function both intrinsically and indirectly through tumor–immune cell interactions. METTL14-dependent m^6^A modification in TAMs can induce CD8^+^ T-cell exhaustion by regulating EBI3 expression (137). m^6^A modification of *PDCD1* mRNA may directly affect T-cell exhaustion and immunotherapy response (138). In tumor cells, regulators such as METTL3, RBM15, IGF2BP1, and NAT10 suppress CD8^+^ T-cell infiltration and cytotoxicity by controlling metabolic programs and immunosuppressive molecules (130, 139–141). These mechanisms suggest that RNA modifications contribute to T-cell dysfunction through both immune-cell-intrinsic regulation and tumor-derived suppressive signals. However, the effects of RNA modifications on immune cells are not uniformly immunosuppressive. In some settings, inhibition of METTL3 enhances interferon responses and antigen presentation, thereby increasing tumor immunogenicity and CD8^+^ T-cell cytotoxicity (142). Conversely, regulators such as YTHDF2 and FTO can promote immune escape by modulating immune checkpoints, glycolytic metabolism, and suppressive immune pathways (106, 143). Pseudouridine may reduce RNA immunogenicity by limiting endogenous double-stranded RNA accumulation, whereas inhibition of pseudouridylation can activate innate immunity and enhance T-cell-mediated antitumor responses (144). These findings highlight the bidirectional and context-dependent nature of RNA modification-mediated immune regulation. Overall, RNA epitranscriptomic modifications reshape immune-cell function through three major mechanisms: first, by regulating myeloid-cell recruitment and suppressive polarization; second, by controlling T-cell differentiation, activation, metabolism, and exhaustion; and third, by coordinating tumor-cell-derived metabolic and checkpoint signals that indirectly impair immune-cell function. In addition, the same regulator may have distinct functions in tumor cells and immune cells, making cell-type-specific models essential. Future studies should integrate RNA modification mapping, single-cell and spatial profiling, and functional immune assays to define causal RNA modification circuits that regulate immune-cell remodeling and immunotherapy response.

### 5.4 Driving metabolic immunosuppression

RNA epitranscriptomic modifications can remodel the tumor immune microenvironment by regulating glycolysis, lactate accumulation, angiogenesis, and immune checkpoint expression (145, 146). These findings indicate that RNA modifications not only control tumor-intrinsic gene expression but also connect metabolic reprogramming with immune suppression. The m^6^A demethylase FTO is an important regulator of metabolism-driven immune evasion. FTO upregulates transcription factors such as *c-Jun, JunB, and C/EBPβ* through m^6^A demethylation, thereby enhancing tumor cell glycolysis (143). FTO knockdown reduces glycolytic activity, restores CD8^+^ T-cell function, and inhibits tumor growth. Moreover, the FTO inhibitor Dac51 synergizes with immune checkpoint blockade, suggesting that targeting FTO may improve immunotherapy efficacy by reprogramming tumor metabolism (143). NAT10-mediated ac^4^C modification also promotes metabolism–immunity crosstalk. In cervical cancer, HOXC8 activates NAT10 expression. NAT10 enhances ac^4^C modification and translation efficiency of *FOXP1* mRNA, which further upregulates *GLUT4 and KHK*, promoting glycolysis and lactate secretion (90). The lactate-enriched TME enhances the immunosuppressive activity of Tregs, whereas NAT10 knockdown improves the therapeutic efficacy of PD-L1 blockade. These findings indicate that the NAT10/ac^4^C/FOXP1 axis links glycolytic reprogramming to immune escape (90). The m^5^C writer NSUN2 promotes glycolysis and histone lactylation in clear cell renal cell carcinoma by stabilizing *NEO1* mRNA. This process further increases PD-L1 expression through the *MYC/POM121/CD274* axis (147). NSUN2 knockdown enhances CD8^+^ T-cell killing and increases TNF-α^+^ T-cell infiltration, suggesting that NSUN2 promotes PD-L1-mediated immune escape through m^5^C-dependent metabolic reprogramming (147). In addition, METTL1-mediated m^7^G modification can enhance *PKM2* expression, promote glycolysis, and induce immunosuppressive signals such as CD155 (148). YTHDF2 may also promote *ETV5* translation and upregulate VEGFA and PD-L1, thereby coordinating angiogenesis and immune escape (149). Overall, metabolic remodeling is a key bridge linking RNA epitranscriptomics to tumor immune evasion. m^6^A, ac^4^C, m^5^C, and m^7^G regulators can promote glycolysis, lactate accumulation, histone lactylation, angiogenesis, and PD-L1/CD155 expression. These changes suppress CD8^+^ T-cell function, enhance Treg-mediated immunosuppression, and establish a metabolism-driven immunosuppressive niche.

### 5.5 Crosstalk among RNA modifications, chromatin regulation, metabolism, and immune signaling pathways

Multiple RNA modifications rarely function in isolation, and accumulating evidence suggests that they may interact cooperatively or antagonistically to shape RNA fate and immune-related gene expression. In some contexts, one modification may facilitate the deposition or recognition of another, whereas in others, distinct modification pathways may compete for shared RNA substrates or recruit functionally opposing reader proteins (15). Such crosstalk may provide an additional layer of regulatory plasticity, allowing tumor and immune cells to dynamically adjust inflammatory signaling, antigen presentation, immune checkpoint expression, and metabolic adaptation. Therefore, future studies should move beyond single-modification-centered models and investigate how coordinated RNA modification networks are established, interpreted, and remodeled during tumor progression and therapeutic intervention (150). Although individual RNA modifications are often discussed separately, accumulating evidence suggests that they operate within interconnected regulatory networks rather than as isolated events. Different RNA modifications may act cooperatively or antagonistically on the same transcript or within the same signaling pathway to fine-tune RNA stability, splicing, localization, translation, and decay (151). In some contexts, one modification may facilitate the deposition or recognition of another modification, thereby reinforcing a specific RNA fate. In contrast, distinct modification pathways may also compete for shared RNA substrates or recruit functionally opposing reader proteins, leading to divergent biological outcomes (20). Such cooperative or antagonistic interactions may help explain why the same RNA modification regulator can exert context-dependent effects in different tumor types, immune-cell states, or therapeutic settings.

RNA modification networks are also closely connected with chromatin regulation and cellular metabolism. Epitranscriptomic regulators can influence the expression of chromatin-modifying enzymes and thereby indirectly shape DNA methylation, histone modifications, chromatin accessibility, and immune-related transcriptional programs (152). Conversely, chromatin states may regulate the transcription of RNA modification writers, erasers, and readers, forming feedback loops between epigenetic and epitranscriptomic control. Metabolism provides an additional layer of regulation because many RNA and chromatin modifications depend on shared metabolites or cofactors, including S-adenosylmethionine, acetyl-CoA, α-ketoglutarate, NAD^+^, and FAD (153). Therefore, metabolic reprogramming in tumor cells and immune cells may alter RNA modification landscapes, while RNA modifications can reciprocally regulate metabolic gene expression, hypoxia adaptation, lactate production, and nutrient stress responses.

Importantly, RNA modifications are integrated with key immune and stress-responsive signaling pathways, including IFN signaling, NF-κB activation, and hypoxia/HIF pathways. Through effects on RNA stability and translation, RNA modifications may modulate the expression of interferon-stimulated genes, inflammatory mediators, antigen-presentation machinery, immune checkpoints, and cytokine networks (98, 154, 155). Similarly, inflammatory signaling pathways may alter the expression or activity of RNA modification regulators, thereby reshaping the epitranscriptomic state during tumor progression or therapy-induced immune remodeling. Under hypoxic conditions, HIF-driven metabolic adaptation may further interact with RNA modification programs to promote immune suppression, angiogenesis, and resistance to therapy (156). Together, these multilayered interactions suggest that RNA modifications should be viewed as part of a broader regulatory network linking RNA fate, chromatin state, metabolism, and immune signaling. Future studies integrating epitranscriptomic profiling with chromatin, metabolomic, and functional immune analyses will be essential to define how these pathways cooperate to shape tumor immune evasion and therapeutic response.

## 6 Clinical translation of RNA epitranscriptomics: biomarkers versus therapeutic targets

### 6.1 Biomarker development

Aberrant RNA modifications are not only involved in tumor progression but may also serve as important biomarkers reflecting tumor immune status and therapeutic response. With the development of multi-omics technologies, regulators of RNA modifications, including m^6^A, m^5^C, m¹A, and ac^4^C, have been systematically characterized across multiple cancer types. Their expression patterns are closely associated with patient prognosis, tumor immune microenvironment status, and sensitivity to immunotherapy. Therefore, molecular subtyping and risk-score models based on RNA modification regulators are emerging as useful tools in precision oncology (157). Several studies have shown that expression signatures of RNA modification regulators can be used for tumor classification and prognostic prediction. For example, in ovarian cancer, high expression of *CBLL1, FTO, METTL3, METTL14, and WTAP* is associated with poorer overall survival and can define distinct immune subtypes (158). m^6^A-related risk models can stratify patients into high- and low-risk groups, with low-risk patients often showing higher immune-cell infiltration and better predicted responses to immune checkpoint therapy (159, 160). Furthermore, scoring systems integrating m^6^A, m^5^C, and m¹A features have shown relatively stable predictive performance across different cohorts. A low RNA modification score is usually associated with a higher immunophenoscore, stronger antitumor immune activity, and greater potential benefit from immunotherapy (161). RNA modification-related biomarkers also show clinical value in specific cancer types. In melanoma, ac^4^C-related models, such as acRGS or AGS, can predict patient survival and distinguish immune “hot” from immune “cold” tumors (162). These scores are closely associated with immune checkpoint expression, tumor mutation burden, and immune-cell infiltration. In lung adenocarcinoma, m^5^C- and m^6^A-related gene signatures, including *HNRNPA2B1, IGF2BP2, NSUN4, and ALYREF*, can be used for prognostic stratification and prediction of immunotherapy response (163). High-risk patients often exhibit increased immune checkpoint expression and higher mutation burden, suggesting that they may benefit from immunotherapy. Similarly, in adrenocortical carcinoma, m^6^A modification patterns are closely associated with immune infiltration and clinical outcomes (163).

Mechanistically, the predictive value of RNA modification-related biomarkers is closely linked to their functions in regulating the tumor immune microenvironment. RNA modifications can control the stability and translation efficiency of immune checkpoint transcripts such as PD-L1, influence immune-cell infiltration, modulate inflammatory signaling pathways, and reshape tumor metabolism. Interactions among different modifications, such as m^6^A, m^5^C, and ac^4^C, further increase regulatory complexity and provide a rationale for constructing multidimensional predictive models. Therefore, integrated models incorporating multiple RNA modification features may improve the accuracy of prognostic assessment and immunotherapy response prediction. In addition to tissue-derived expression signatures, RNA modifications in blood and extracellular vesicles may have potential as liquid biopsy biomarkers (164, 165). Modification changes in circulating RNA, plasma RNA, or exosomal RNA may reflect tumor burden, immune status, and therapeutic response. These biomarkers could be used for dynamic monitoring during immunotherapy, early detection of resistance, and longitudinal evaluation of tumor immune microenvironment remodeling. However, several limitations remain. Most RNA modification-related signatures are derived from retrospective analyses of public datasets and lack large-scale prospective clinical validation. Detection methods, scoring systems, and clinical thresholds have not yet been standardized. Reproducibility across cancer types, patient cohorts, sequencing platforms, and bioinformatic pipelines remains limited. Therefore, although RNA modification-related biomarkers show promising value for prognosis evaluation and immunotherapy prediction, their clinical translation still requires further experimental validation, prospective cohort studies, and standardized detection systems.

### 6.2 Therapeutic targeting of RNA modification regulators

RNA epitranscriptomic modifications represent an important post-transcriptional regulatory layer in tumor progression and immune regulation (75). Because RNA modifications are dynamic and partially reversible, targeting their writers, erasers, and readers has emerged as a promising anticancer strategy (75). Compared with conventional gene- or protein-targeted approaches, RNA modification regulators can simultaneously affect RNA stability, translation efficiency, metabolic reprogramming, tumor stemness, and immune evasion. Therefore, these regulators may suppress tumor growth and enhance sensitivity to immunotherapy. However, it should be emphasized that most RNA modification-targeting drugs remain in preclinical or early translational stages, and their clinical efficacy, safety, and patient selection criteria have not been fully established. Targeting m^6^A erasers is one of the most actively investigated strategies. FTO acts as an oncogenic factor in multiple cancers. Its inhibitor FB23–2 restores m^6^A levels, suppresses leukemia stem cell self-renewal, and induces differentiation and apoptosis (166). In addition, FTO inhibition can enhance T-cell-mediated cytotoxicity by downregulating immune checkpoint-related molecules such as LILRB4, thereby reversing immune evasion (166). Another FTO inhibitor, Dac51, has been reported to block FTO-mediated metabolic immune escape and synergize with immune checkpoint blockade (143). Similarly, ALKBH5 inhibitors such as W23–1006 increase m^6^A levels on target RNAs and suppress tumor cell proliferation, migration, and metastasis (167). These findings suggest that targeting m^6^A erasers may affect both tumor-intrinsic malignant phenotypes and the immune microenvironment.

Targeting RNA modification writers also shows antitumor potential. METTL3 is a core component of the m^6^A methyltransferase complex (168). Its inhibitor STM2457 reduces m^6^A modification on oncogenic transcripts, suppresses acute myeloid leukemia cell proliferation, induces differentiation and apoptosis, and selectively eliminates leukemia stem cells (168). NAT10 is an ac^4^C writer that promotes metabolic adaptation and tumor progression by enhancing RNA stability and translation efficiency (50). NAT10 inhibitors, such as paliperidone and AG-401, can block metabolic pathways such as the ATF4-ASNS axis and inhibit tumor growth (80). Because writers act upstream of RNA modification deposition, their inhibition may broadly affect multiple downstream transcripts and signaling networks. This provides strong therapeutic potential but also raises major concerns regarding safety and specificity. Targeting reader proteins may directly block the functional output of RNA modification signals. For example, YTHDF1 promotes the translation of m^6^A-modified mRNAs and supports tumor cell proliferation. Its inhibitor tegaserod disrupts YTHDF1 binding to target mRNAs, reduces the expression of key cell-cycle regulators, and suppresses tumor progression (169). IGF2BP2 stabilizes mRNAs involved in metabolism and stemness, and its inhibitor CWI1–2 significantly suppresses tumor growth (170). The nuclear reader YTHDC1 is also a potential therapeutic target. Compounds such as YL-5092 can reduce the stability of specific mRNAs, induce apoptosis, and eliminate leukemia stem cells (171). These studies indicate that reader proteins are key execution nodes linking RNA modifications to biological outcomes, and targeting readers may more directly interrupt oncogenic RNA regulatory programs.

Despite these encouraging findings, the clinical translation of RNA modification-targeted therapy faces several major challenges. First, many inhibitors have only been tested *in vitro*, in animal models, or in early drug-development settings, and evidence from clinical trials remains limited. Second, RNA modification enzymes are broadly involved in normal hematopoiesis, immune-cell differentiation, development, and tissue homeostasis; therefore, systemic inhibition may cause potential toxicity. Third, drug specificity remains a critical issue, because some small molecules may have off-target effects, and the same enzyme may act on multiple RNA substrates or even different RNA modifications. Fourth, RNA modification regulators exhibit strong cancer type-, cell type-, and microenvironment-dependent functions. For example, FTO, METTL3, or METTL14 may exert opposite effects in different tumors, and thus cannot be simply regarded as universal oncogenic targets (93, 172, 173). Fifth, reliable biomarkers for patient selection are still lacking. It remains unclear which patients are truly dependent on FTO-, METTL3-, NAT10-, or IGF2BP2-related pathways. Future development of RNA modification-targeted therapy should therefore focus on three major directions. First, highly selective inhibitors with low toxicity should be developed. Second, the cell-type-specific functions of RNA modification regulators in tumor cells and immune cells must be clarified. Third, patient stratification strategies based on RNA modification signatures, target-gene expression, and immune microenvironment features should be established. In particular, combination strategies integrating RNA modification inhibitors with immune checkpoint blockade, radiotherapy, chemotherapy, or targeted therapy may improve their clinical value. Overall, targeting RNA modification regulators has strong translational potential, but it should currently be considered an emerging therapeutic field rather than a mature clinical strategy.

### 6.3 targeting of RNA modification regulators combination with immunotherapy

**Figure 2:**
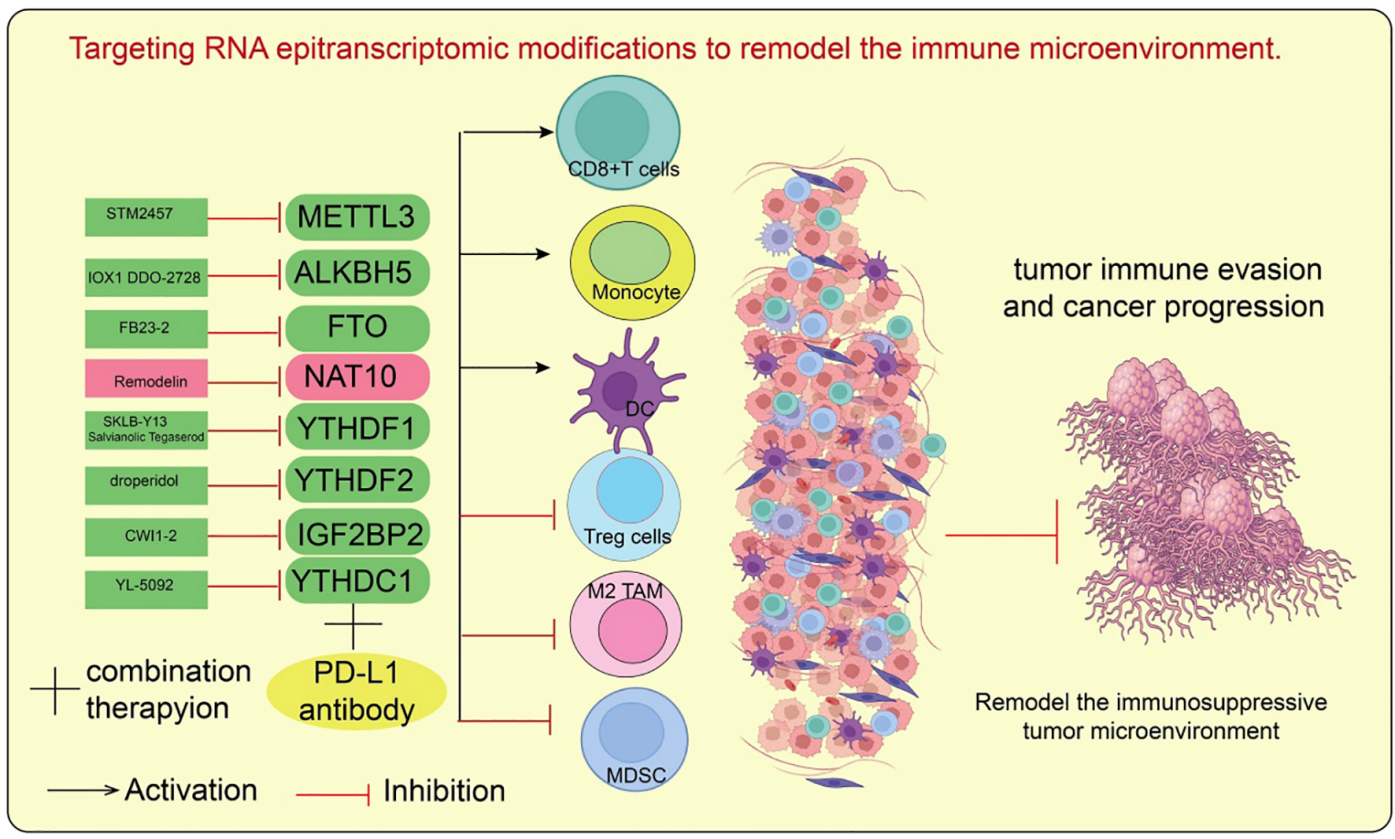
Targeting RNA epitranscriptomic modifications to remodel the tumor immune microenvironment. Schematic illustration of pharmacological targeting of key RNA epitranscriptomic regulators and their impact on the tumor immune microenvironment (TME). Small-molecule inhibitors, including STM2457 (METTL3 inhibitor), IOX1 and DDO-2728 (ALKBH5 inhibitors), FB23-2 (FTO inhibitor), Remodelin (NAT10 inhibitor), SKLB-Y13, salvianolic acid, and tegaserod (YTHDF1 inhibitors), droperidol (YTHDF2 inhibitor), CW1-2 (IGF2BP2 inhibitor), and YL-5092 (YTHDC1 inhibitor), modulate the activity of RNA modification “writers,” “erasers,” and “readers.” Targeting these factors enhances antitumor immunity by promoting CD8^+^ T cell activation, facilitating monocyte and dendritic cell (DC) function, and suppressing immunosuppressive cell populations, including regulatory T (Treg) cells, M2 tumor-associated macrophages (TAMs), and myeloid-derived suppressor cells (MDSCs). Combination therapy with PD-L1 blockade further potentiates these effects, leading to remodeling of the immunosuppressive TME, inhibition of tumor immune evasion, and suppression of cancer progression.

RNA modifications not only regulate tumor development but also play critical roles in tumor immune evasion (174). Their combination with immunotherapy, especially immune checkpoint inhibitors such as PD-1 or PD-L1 antibodies, has become a promising strategy to improve response rates and overcome resistance (109, 174). RNA modifications regulate antigen presentation, immune cell infiltration, recruitment of immunosuppressive cells, and immune checkpoint expression, thereby determining tumor sensitivity to immunotherapy. At the level of antigen presentation and T cell activation, the m6A reader YTHDF1 acts as a key negative regulator. YTHDF1 enhances translation of lysosomal proteases, accelerates antigen degradation, and suppresses cross-presentation by dendritic cells, thereby limiting CD8^+^ T cell activation (175). Its depletion enhances antigen presentation and antitumor immunity and significantly improves the efficacy of anti PD-L1 therapy (175). In colorectal cancer, YTHDF1 also promotes p65 translation, upregulates CXCL1, drives recruitment of MDSCs, and suppresses CD8^+^ T cell function. Targeting YTHDF1 reduces MDSC infiltration, enhances T cell cytotoxicity, and overcomes resistance to anti PD-1 therapy (81). In **Figure 2**, we illustrates how targeting RNA epitranscriptomic regulators reshapes the tumor immune microenvironment and enhances antitumor immunity, particularly in combination with PD-L1 blockade.

RNA modifications also directly regulate immune checkpoint expression. METTL3 promotes m6A-dependent decay of PD-L1 mRNA. Its inhibition increases PD-L1 expression and enhances CD8^+^ T cell infiltration, thereby improving the efficacy of PD-1 blockade (176). Similarly, ALKBH5 maintains PD-L1 stability through demethylation and suppresses T cell function, whereas its depletion promotes *PD-L1* degradation and enhances immune responses (177). NSUN2-mediated m5C modification and IGF2BP2-mediated mRNA stabilization also sustain high *PD-L1* expression and promote T cell exhaustion (112, 178). Targeting these regulators reduces PD-L1 levels and improves immunotherapy outcomes. RNA modifications also reshape the tumor immune microenvironment. YTHDF2 is upregulated after radiotherapy and promotes MDSC expansion, forming an immunosuppressive feedback loop (98, 102). Its inhibition reverses immune suppression and enhances the efficacy of radiotherapy combined with PD-L1 blockade. In tumor associated macrophages, YTHDF2 suppresses antitumor polarization, and its inhibition enhances antigen presentation and CD8^+^ T cell activity (98, 179).

RNA modification also links metabolic reprogramming to immune suppression. NAT10-mediated Ac4C modification enhances glycolysis and lactate accumulation, thereby promoting regulatory T cell mediated immunosuppression (90). Its inhibition reduces immune suppression and improves PD-L1 blockade efficacy. Similarly, METTL1 and IGF2BP2 regulate lipid metabolism and membrane dynamics, influencing PD-L1 localization and stability and promoting immune evasion. Targeting these pathways improves the immune microenvironment and enhances therapeutic responses (111, 180). RNA modification also synergizes with other treatments such as radiotherapy and CAR-T therapy. YTHDF2 promotes metabolic adaptation and antigen escape in B cell malignancies, and its inhibition enhances CAR-T efficacy (106). In radiotherapy, targeting YTHDF2 overcomes therapy-induced immune suppression and improves combination treatment outcomes. Overall, RNA modifications regulate multiple aspects of tumor immunity and represent promising targets to enhance immunotherapy efficacy and overcome resistance.

Although targeting RNA modification regulators represents a promising strategy to enhance antitumor immunity and improve responses to immunotherapy, the translational maturity of this field remains uneven. Most current evidence is derived from cell-based experiments, animal models, or retrospective analyses of patient cohorts, whereas clinically validated therapeutic strategies directly targeting RNA modification machinery in combination with immune checkpoint blockade are still limited. Therefore, RNA modification regulators should be considered at different levels of evidence: some serve as experimentally supported modulators of immune phenotypes in preclinical models, some represent emerging biomarkers or therapeutic hypotheses requiring prospective validation, and only a limited number have entered early-stage translational or clinical investigation. Moreover, the therapeutic effects of these regulators are often context-dependent, varying according to tumor type, cellular compartment, immune microenvironment, and treatment status. Future studies should therefore combine mechanistic validation, biomarker-driven patient stratification, and well-designed clinical trials to determine whether targeting RNA epitranscriptomic pathways can be safely and effectively integrated with immunotherapy.

## 7 Future perspectives

Although the roles of RNA modifications in tumor biology and immune regulation are increasingly recognized, several challenges remain in their basic study and clinical translation. One major limitation is insufficient specificity. RNA modifications such as m6A, m5C, and Ac4C act at the transcriptome-wide level, and their writers, erasers, and readers regulate a large number of mRNAs. While this broad regulation confers strong biological effects, it also increases the risk of off-target effects. Targeting enzymes such as METTL3 or FTO may affect hundreds of transcripts and disrupt normal cellular homeostasis (181). Achieving transcript-specific or cell type specific regulation will be a key goal for future drug development. Another challenge is tumor heterogeneity. Different cancer types, and even different subtypes within the same cancer, show substantial variation in RNA modification enzyme expression, target selection, and functional output. The same regulator may promote immune evasion in one context but enhance immune activation in another. These context dependent effects highlight the influence of the tumor microenvironment, metabolic state, and genetic background. Large scale multi-omics studies will be needed to stratify tumors and guide precise therapeutic strategies.

Crosstalk among RNA modifications further increases regulatory complexity. Different modifications such as m6A, m5C, m1A, Ac4C, and pseudouridine do not act independently but interact through synergistic or antagonistic mechanisms (58). Individual transcripts may carry multiple modifications that collectively influence stability, splicing, or translation. Enzymes may also share substrates or regulate common pathways. This complex epitranscriptomic network remains incompletely understood and limits comprehensive mechanistic insights. In clinical translation, toxicity and delivery remain major challenges. Because RNA modifications are essential for normal physiology, systemic inhibition may cause adverse effects. In addition, the stability, targeting efficiency, and delivery of small molecules or RNA based therapeutics require further optimization. Effective delivery to tumor cells or specific immune populations such as T cells or TAMs is particularly challenging in solid tumors (182). Development of highly selective inhibitors, controlled release systems, and targeted delivery platforms such as nanoparticles will be critical. Emerging technologies such as single cell RNA sequencing and spatial transcriptomics provide new opportunities. Traditional bulk sequencing cannot resolve cellular heterogeneity within the tumor microenvironment. High resolution approaches can reveal cell type specific and spatially resolved RNA modification patterns. Integration of RNA modification profiling with single cell and multi-omics data will enable systematic mapping of regulatory networks and identification of precise therapeutic targets and biomarkers. Overall, advancing specificity, understanding crosstalk, overcoming delivery barriers, and leveraging high resolution technologies will drive the clinical application of RNA modification based therapies.

Despite rapid advances in single-cell RNA sequencing, spatial transcriptomics, and epitranscriptomic profiling technologies, several methodological limitations remain important when interpreting RNA modification studies. Current approaches for mapping RNA modifications, including antibody-based enrichment methods, chemical-labeling strategies, nanopore direct RNA sequencing, and mass spectrometry, each have distinct strengths and limitations. Antibody-based methods can identify transcriptome-wide modification-enriched regions but often lack single-nucleotide resolution and may be affected by antibody specificity and enrichment bias. Chemical and enzyme-assisted methods improve site resolution for selected modifications but may introduce conversion efficiency or sequence-context biases. Nanopore sequencing offers the possibility of direct RNA modification detection, yet its accuracy and quantitative reliability still require further optimization and orthogonal validation. In addition, most mapping methods provide relative enrichment rather than absolute modification stoichiometry, making it difficult to determine what fraction of transcripts is modified at a given site. Because RNA modification levels can vary across cell types, tumor regions, treatment states, and immune compartments, future studies should integrate multiple complementary technologies with functional assays to distinguish correlative modification changes from causally relevant regulatory events.

## 8 Conclusion

RNA epitranscriptomic modifications act as a critical bridge between gene regulation and tumor immune responses and represent a key regulatory layer in tumor immune evasion. The key message of this review is that RNA epitranscriptomic modifications constitute a multilayered regulatory system that connects RNA fate with tumor immune evasion. Through writers, erasers, and readers, RNA modifications regulate immune-related transcript stability, translation, splicing, export, and degradation. These molecular effects converge on four major biological processes: tumor immune visibility, checkpoint and inflammatory signaling, immune-cell remodeling, and metabolic reprogramming of the tumor microenvironment. Importantly, RNA modifications can either promote immune escape or enhance antitumor immunity depending on cancer type, cellular context, target transcript, and reader protein. Therefore, future therapeutic strategies should not simply inhibit or activate a single RNA modification enzyme, but should consider the context-specific RNA modification network and its interaction with tumor immunity.
